# Factor analysis of self-treatment in diabetes mellitus: a cross-sectional study

**DOI:** 10.1186/1471-2458-11-761

**Published:** 2011-10-04

**Authors:** Negin Masoudi Alavi, Leila Alami, Sedigheh Taefi, Gholamali Shojae Gharabagh

**Affiliations:** 1 Faculty of Nursing, Kashan University of Medical Sciences, Ghotb Ravndi Highway, Kashan, IRAN

**Keywords:** Diabetes Mellitus, Self-Treatment, Factor analysis

## Abstract

**Background:**

Self-treatment is a treatment of oneself without professional help, which may cause health-related consequences. This investigation examined the self-treatment behaviors in patients with diabetes mellitus in Iran/kashan.

**Methods:**

The patients who referred to the clinic of diabetes and those who were admitted to the General hospital in the city of Kashan due to diabetes mellitus were asked to participate in this cross-sectional study. For data collection, The 25 item questionnaire of Likert scale type with four scales was used. Factor analysis was performed to define the patterns of self-treatment.

**Results:**

398 patients participated in the study. The mean age of the study population was 54.9 ± 12.9 years. The majority (97%) had type 2 diabetes. 50% of patients reported self- treatment. The self-treatment score was 45.8 ± 8.8 (25-100). Female gender, lower education and co-morbid illnesses of hypertension, hyperlipidemia and cardiac disease had significant relationship with self-treatment. The factor analysis procedure revealed seven factors that explained the 43% of variation in the self-treatment. These seven factors were categorized as knowledge, deficiencies of formal treatments, available self-treatment methods, physician related factors, the tendency to use herbal remedies, underlying factors such as gender and factors related to diabetes.

**Conclusions:**

There is a medium tendency for self-treatment in diabetic patients. The assessment of self-treatment practices must be an essential part of patients' management in diabetes care.

## Background

Self treatment is a treatment of oneself without professional help, to alleviate an illness or a condition [1]. Persons with a strong self-treatment tendency might not seek any formal treatment [2]. Self-treatment is often associated with adverse effects, related to the improper use of substances [3]. Self-treatment is a common behavior all over the world. It is estimated that 83.3% of patients commit self-treatment in Iran [4]. In a study, the 75% of participants were reported to take analgesics for pain management, which were successful only in 45% of cases [5]. The symptom iceberg means that the majority of symptoms were self-treated. It is estimated that only one symptom in 37 led to a consultation [6].

Diabetes mellitus contributes to the increased morbidity and mortality [7]. Considering the burden of the disease, self-treatment might have severe consequences [8]. In a case report, a diabetic patient tried to treat the foot injury by using a hot paraffin footbath, but severe burn injury developed and after surgical debridement and a long healing period of six months, the patient recovered [9]. Self-treatment in diabetes is generally considered to be safe. Using herbal remedies or bush medicines for treating the symptoms of diabetes is common [8]. Even in some cases such as symptomatic hypoglycemia, self-treatment might be life-saving [10].

Self-treatment is a multidimensional construct involving complex processes, which influence personal and public health. Income, ethnicity, educational level, age, cultural forces and health perspectives are among the factors that might influence this behavior [3]. At present, little is known about the self-treatment behaviors in diabetes mellitus [11]. The aim of the current study was to assess the prevalence of self-treatment behaviors in diabetes mellitus in Kashan and to determine the variables and patterns that could influence this behavior. The cross-sectional research method and factor analysis were used for this study.

## Methods

In this cross-sectional study, all the patients who referred to the clinic of diabetes in Kashan and those who were hospitalized in General hospital of the city due to diabetes mellitus during January to June 2009 were asked to participate in the study. The inclusion criteria for this study were as follows: age above 18 years old and diagnosed with diabetes mellitus by Glucose Tolerance Test for at least six months. 398 of the total 420 patients accepted to take part in the study. The difference between the patients who accepted and those who refused to participate in this study were not remarkable. The research was approved by the ethical committee of Kashan University of Medical Sciences, and the informed consent was obtained from each subject.

The items of the questionnaire were extracted from the interviews in a qualitative research. The self-treatment experiences that were quoted by patients were changed to the items of the questionnaire. The details of this research have been described elsewhere [12]. Item analysis of the questionnaire was carried out in a pilot study. 40 patients completed the questionnaire in the pilot study. The Cronbach Coefficient Alpha of the questionnaire was calculated in the pilot study. Considering the findings, 10 items with low internal consistency were dropped. The final questionnaire contained 25 items with a 4-point Likert scale ranging from always to never. Every item represented a self-treatment behavior or the possible factors that could influence it, such as "I use herbal remedies or bush medicines for treating diabetes " or "I believe the herbs are harmless." The internal consistency reliability of the questionnaire was 0.85. The content validity of the questionnaire was confirmed by the experts in the diabetes field. The patients could obtain a score between 25 and 100 from the questionnaire. The higher scores indicated the greater self-treatment tendency. The raw score was used for data analysis.

The variable including age, sex, type of diabetes, modes of treatment, education, duration of diabetes and co-morbid illnesses were also recorded. The patients completed the questionnaire in the presence of an interviewer, who intervened if there were any difficulties in reading and understanding the questions.

The data was compiled and statistically analyzed using the 16.0 release of SPSS program for Windows. The relationships between variables and self-treatment scores were analyzed by Kruskal-Wallis and Mann-Whitney test. Analysis of covariance used to adjust for possible confounders. To explore the self-treatment behaviors and its related variables, exploratory factor analysis was conducted using the maximum likelihood technique with varimax rotation. The items and variables were assigned to the composite scale on which it had the highest factor loading. The Kaiser-Mayer-Olkin (KMO) measure and Bartlett's test of sphericity were conducted to find sampling adequacy. The goals of exploratory factor analysis were to determine the number of fundamental influences underlying a domain of variables, to quantify the extent to which each variable was associated with the factors.

## Results

### Sample characteristics

398 of 420 patients participated in the study. The mean age of the study population was 54.9 ± 12.9 years. The patients had 9.5 ± 8.3 years, history of diabetes. The majority (97%) had type 2 diabetes and 273 (68.5%) were female. The 47.7% of patients had hypertension and 27.6% had hyperlipidemia. The 32.4 percent of patients were treated by insulin injections while 67.6% of patients received oral drugs. The socio-demographic characteristics of subjects and their relationship with self-treatments are presented in Table 1.

**Table 1:** The characteristics of the patients and its relationship with self-treatment

		N	%	Self- treatment Score	Mean Rank	P value	Covariant	
							
							Age	Education
Gender	Female	273	68.5	46.8 ± 8.6	214.5	0.0001*	F = 3.38	F = 0.974
				
	Male	125	31.5	43.7 ± 8.9	170.4		p = 0.066	p = 0.324

Treatment	Insulin	129	32.4	46.7 ± 8.4	208.16	0.2		
				
	Oral pills	269	67.6	45.4 ± 8.9	192.43			

Kind of referring	Inpatient	198	49.7	46.5 ± 8.7	210.03	0.099		
				
	Outpatient	200	50.3	45.1 ± 8.9	190.97			

Education	Illiterate	174	43.7	47.1 ± 8.4	217.53	0.014*	F = 0.172	
				
	Under diploma	176	44.3	44.9 ± 9.2	183.55		p = 0.678	
				
	Diploma & higher	48	12	44.7 ± 8.6	183.7			

Renal problems Θ	Yes	82	20.8	45.4 ± 9.3	194.22	0.578		
				
	No	316	79.2	45.9 ± 8.7	202.15			

Hyperlipidemia ¥	Yes	110	27.6	47.4 ± 8.8	220.18	0.035*	F = 1.48	F = 3.15
				
	No	288	72.4	45.2 ± 8.7	192.94		p = 0.224	p = 0.076

Cardiac disease ¶	Yes	133	33.4	47 ± 8.6	217.25	0.04*	F = 1.06	F = 3.09
				
	No	265	66.6	45.2 ± 8.9	192.06		p = 0.3	p = 0.08

Hypertension δ	Yes	190	47.7	47.1 ± 9.1	216.9	0.007*	F = 0.57	F = 2.5
				
	No	208	52.3	44.6 ± 8.5	185.52		p = 0.45	p = 0.116

*Significant relationship

Θ Glumerular Filtration Rate less than 60 mL/min/1.73m2

¥ Cholestrol more than 200 mg/dL

¶ The symptoms of angina pectoris according to physician diagnosis

δ Sistolic blood pressure above 140 mmHg or diastolic pressure above 90 mmHg

### Self-treatment behaviors

23% of patients chose always or most of the time for practicing self-treatment. 27% chose sometimes and 50% selected never for self-treatment practice. The self-treatment score was 45.8 ± 8.8 (Minimum 25, Max 75, Median = 45, Mode = 39). Female gender, lower education and co-morbid illnesses of hypertension, cardiac disease and hyperlipidemia showed significant relationship with self-treatment. The self-treatment score in patients who referred to the outpatient clinics and those who were hospitalized was not significantly different. Using analysis of covariance to adjust for age and education did not modify the results (Table 1).

Self-medication was a common form of self-treatment (27%). Using herbs, plants and opium were other forms of self-treatment (12.2%). The patients tended to use self-treatment for symptoms such as fatigue and pain and to reduce the blood-glucose level. Most patients seek formal treatments (54.3%) after their unsuccessful self-treatment and when they face severe problems following chronic and acute complications of diabetes such as hypoglycemia or renal problems.

### Factor analysis

**Figure 1:**
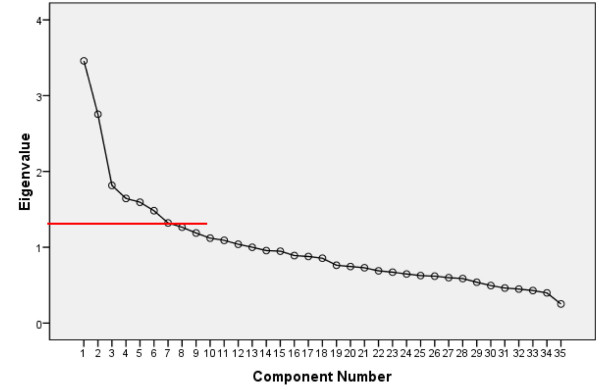
Scree Plot of eigenvalues of self-treatment variables

This study showed the KMO of 0.618 and the Bartlett's test of sphericity of 0.000 (less than 0.05), making the sample of this study adequate for factor analysis. From scree plot levels off to a linear decreasing pattern, seven major factors were elicited (Figure 1).

These seven factors were named: Knowledge, deficiencies of formal treatments, available self-treatment methods, physician related factors, tendency to use herbal remedies, underlying factors such as gender, and factors related to diabetes. The accumulated variance reached 43% (Table 2).

**Table 2:** Summary of factors' structures

Factors	Factors' Name	Variables	Loading	Eigenvalue	Percent of variance	Accumulated Variance (%)
Factor 1	Knowledge	General Education	0.433	3.46	10%	10%
					
		Age	0.932			
					
		Diabetes related education	0.422			

Factor 2	Deficiencies of formal treatments	Fears of treatments	0.327	2.75	8%	18%
					
		Long stay for visiting a physician	0.405			
					
		Costly treatments	0.520			
					
		Frustration of treatment	0.611			

Factor 3	Available self-treatment methods	Home remedies	0.922	1.81	6%	24%
					
		Doing nothing and waiting to see the progress	0.587			
					
		Self-medication	0.371			
					
		Use of herbals	0.352			

Factor 4	Physician related factors	Easy physician consultation	0.358	1.64	5%	29%
					
		Believing physician's competencies	0.452			
					
		Respect of physician	0.355			
					
		Tendency to changing physician	0.437			
					
		Having several physicians	0.464			

Factor 5	Tendency to use herbal remedies	Believing effectiveness of herbals	0.507	1.59	5%	34%
					
		Believing Herbals are safe	0.403			

Factor 6	Underlying factors	Female Gender	0.634	1.48	5%	39%
					
		Family support	0.378			
					
		Co-morbid illnesses	0.376			

Factor 7	Factors related to diabetes	Type of Diabetes	0.402	1.32	4%	43%
					
		Duration of diabetes	0.384			
					
		Treatment modality (Insulin, oral pills)	0.576			

## Discussion

In this study, there was a medium tendency for self-treatment among diabetic patients.

Female gender, lower education and co-morbid illnesses of hypertension, cardiac disease and hyperlipidemia showed the relationship with self-treatment. If the treatment with Insulin or renal complications were considered as the indicator of severity of the diabetes, it seems that severity of the diabetes is not significantly related to self-treatment, but the symptoms that patient experience might determine his or her self-treatment behavior. Patients prefer to seek formal treatments in severe symptoms. The inpatient and outpatient situations were not related to self-treatment behaviors. This might represent the fact that self-treatment is a basic health-related behavior that is not affected by temporary situations.

Different factors could influence self-treatment behaviors. Giovannini found that self-treatment is the most common first therapeutic choice in the patients [13]. However, we should consider that self-treatment is a broad concept, and a process which can be divided into three-stages of self-diagnosis, self-treatment and self-monitoring [14]. The patients' treatment behaviors can be ranged from seeking care from doctors as outpatients, waiting to see the progress of symptoms, resting or consulting books, using home remedies to self-treatments [15].

In our study, patients showed different self-treatment behaviors. Self-medication is the most prevalent self-treatment behavior. 27% of patients reported self medication. In Tabriz 81.64% of individuals embarked on self-treatment using chemical drugs [16]. This is much higher compared to our study. It seems that self-treatment is more common in general population than in patients with diabetes mellitus.

The next aim of this study was to identify the background factors of self-treatment in diabetes. The seven factors were extracted from the data. In a study, the most common factors' underlying self-drug consumption was negligence of the disease (30%) and inability to afford the visit fees of the physicians (14.7%) [4]. In a different study in Iran, it was found that costly treatments (32.4%) and lack of time (23.7%) were the reasons why patients refused to visit the physicians [17]. Another study showed that symptomatic therapy by physician, insignificance of diseases from the patients' viewpoints, and cost of the treatments were the main reasons for practicing self-medication [18]. The comparable factors can be seen in our study. Like us, Jirojwong also found that compared to men, women tend more to use home remedies and show more self-treatment behaviors [15]. The women had significantly lower education compared to men, which can explain the difference. But studies show that there are basic differences between men and women in treatment-seeking behaviors and self-treatment [15,12,19,5].

It is possible that the medium tendency of self-treatment in patients to be related to their tendency of not choosing the extreme options in the questionnaire. Convenience sampling was used for this research, and it should be noted that the participants of this study were patients with diabetes seeking care at the ambulatory clinic or admitted to the hospital due to diabetes complications, which might be a potential source of bias and limits the generalization of the findings.

## Conclusion

People make decisions about their methods of treatment based on their experiences, which is formed as a response to particular illness episodes and interactions of many overlapping components. The explosion of information on medicine has enhanced the pluralistic system and patients can choose different self-treatment methods [20].

We need more information about self-treatment behaviors to distinguish its negative and potential positive impacts on the treatment process and to develop better educational programs. Education in diabetes mellitus must be very precise. Patients need to know when they should consult a physician immediately and when they can take action by themselves. We need to know the common self-treatment modalities in our community and predict their hazards and benefits. The assessment of self-treatment practices must be an essential part of patients' management in diabetes care.

## Competing interests

The authors declare that they have no competing interests.

## Authors' contributions

All the 4 authors contributed to research design and data gathering. The corresponding author Dr Negin Masoudi Alavi analyzed the data and wrote the final report and article. All the authors read and approved the final manuscript

## Pre-publication history

The pre-publication history for this paper can be accessed here:

http://www.biomedcentral.com/1471-2458/11/761/prepub
